# Development and Validation of a Deep Learning Model for Brain Tumor Diagnosis and Classification Using Magnetic Resonance Imaging

**DOI:** 10.1001/jamanetworkopen.2022.25608

**Published:** 2022-08-08

**Authors:** Peiyi Gao, Wei Shan, Yue Guo, Yinyan Wang, Rujing Sun, Jinxiu Cai, Hao Li, Wei Sheng Chan, Pan Liu, Lei Yi, Shaosen Zhang, Weihua Li, Tao Jiang, Kunlun He, Zhenzhou Wu

**Affiliations:** 1 National Center for Clinical Medicine of Neurological Diseases, Beijing, People’s Republic of China; 2 Department of Radiology, Beijing Tiantan Hospital, Capital Medical University, Beijing, People’s Republic of China; 3 Department of Neurology, Beijing Tiantan Hospital, Capital Medical University, Beijing, People’s Republic of China; 4 China National Clinical Research Center for Neurological Diseases, Beijing Hanalytics Artificial Intelligence Research Center for Neurological Disorders Beijing, PR China; 5 Medical Imaging Department, Shenzhen Second People’s Hospital/the First Affiliated Hospital of Shenzhen University Health Science Center, Shenzhen, People’s Republic of China; 6 Translational Medicine Laboratory, Chinese People's Liberation Army General Hospital, Beijing, People’s Republic of China; 7 Key Laboratory of Ministry of Industry and Information Technology of Biomedical Engineering and Translational Medicine, Chinese People's Liberation Army General Hospital, Beijing, People’s Republic of China

## Abstract

**Importance:**

Deep learning may be able to use patient magnetic resonance imaging (MRI) data to aid in brain tumor classification and diagnosis.

**Objective:**

To develop and clinically validate a deep learning system for automated identification and classification of 18 types of brain tumors from patient MRI data.

**Design, Setting, and Participants:**

This diagnostic study was conducted using MRI data collected between 2000 and 2019 from 37 871 patients. A deep learning system for segmentation and classification of 18 types of intracranial tumors based on T1- and T2-weighted images and T2 contrast MRI sequences was developed and tested. The diagnostic accuracy of the system was tested using 1 internal and 3 external independent data sets. The clinical value of the system was assessed by comparing the tumor diagnostic accuracy of neuroradiologists with vs without assistance of the proposed system using a separate internal test data set. Data were analyzed from March 2019 through February 2020.

**Main Outcomes and Measures:**

Changes in neuroradiologist clinical diagnostic accuracy in brain MRI scans with vs without the deep learning system were evaluated.

**Results:**

A deep learning system was trained among 37 871 patients (mean [SD] age, 41.6 [11.4] years; 18 519 women [48.9%]). It achieved a mean area under the receiver operating characteristic curve of 0.92 (95% CI, 0.84-0.99) on 1339 patients from 4 centers’ data sets in diagnosis and classification of 18 types of tumors. Higher outcomes were found compared with neuroradiologists for accuracy and sensitivity and similar outcomes for specificity (for 300 patients in the Tiantan Hospital test data set: accuracy, 73.3% [95% CI, 67.7%-77.7%] vs 60.9% [95% CI, 46.8%-75.1%]; sensitivity, 88.9% [95% CI, 85.3%-92.4%] vs 53.4% [95% CI, 41.8%–64.9%]; and specificity, 96.3% [95% CI, 94.2%-98.4%] vs 97.9%; [95% CI, 97.3%-98.5%]). With the assistance of the deep learning system, the mean accuracy of neuroradiologists among 1166 patients increased by 12.0 percentage points, from 63.5% (95% CI, 60.7%-66.2%) without assistance to 75.5% (95% CI, 73.0%-77.9%) with assistance.

**Conclusions and Relevance:**

These findings suggest that deep learning system–based automated diagnosis may be associated with improved classification and diagnosis of intracranial tumors from MRI data among neuroradiologists.

## Key Points

**Question:**

How can deep learning be used for brain tumor classification and diagnosis?

**Findings:**

In this diagnostic study of a deep learning system trained using magnetic resonance imaging data from 37 871 patients, the system performed better than neuroradiologists in classifying and identifying brain tumors. Neuroradiologists had higher accuracy with vs without the aid of the deep learning system.

**Meaning:**

These findings suggest that deep learning may be able to aid neuroradiologists in brain tumor classification and diagnosis.

## Teaser

This diagnostic study investigates tumor diagnosis and classification outcomes with and without use of a deep learning system based on magnetic resonance imaging data.

## Introduction

Intracranial tumors span a broad spectrum of pathologies. The type of the tumor significantly impacts the course of treatment and prognosis. Treatment decisions, including surgery, radiation, and pharmacotherapy, rely on early diagnosis and accurate classification of the tumor.^1,2,3^

Magnetic resonance imaging (MRI) and biopsy are routinely performed for the diagnosis of intracranial tumors, with biopsy considered a criterion standard for the classification of tumor types.^4^ Although a standard practice, biopsy has associated challenges because it is invasive. Therefore, identification and accurate classification of tumor subtypes from noninvasive methods like MRI are desired. However, identification of tumor types, particularly rare types, from MRI data has been challenging because of the similar phenotypes of multiple tumor classes on MRI scans. As an example, distinguishing primary central nervous system (CNS) lymphoma–mimicking glioblastoma from MRI scans is challenging.^5,6,7^ Therefore, an accurate, reliable preoperative determination of tumor types from MRI may facilitate rapid clinical decision-making and aid in better treatment planning.

With the rapid development of radiomics and research in image-processing algorithms in the last 9 years, several methods of image interpretation, particularly deep learning–based methods, have shown a potential utility in the medical domain.^8^ From segmentation studies to radiogenomics studies, the application of modern deep learning systems (DLSs) has been found to be associated with significant advantages in recognizing complex patterns in medicine.^9,10,11,12,13,14,15^ Studies have also explored the use of DLS for tumor segmentation from MRI scans.^16,17^ In 2016, Meier et al^11^ presented a system for brain tumor segmentation. In 2017, Young et al^18^ developed a system for investigating prognostic-relevant subtypes of glioblastoma. Meanwhile, Kang et al^5^ developed a system for the atypical manifestation of primary CNS lymphoma.

However, to the best of our knowledge, no prior study has explored the task of multiclass tumor identification and classification, which includes the most commonly occurring types of tumors, as well as rarer tumor types. Furthermore, most previous studies have been developed with a limited number of patients, and the need for a generalizable system that can achieve good performance on multicenter clinical data remains unmet. Therefore, we investigated the accuracy and timeliness of classification of 18 brain tumor classes by a DLS trained using MRI data from more than 37 000 patients to improve diagnosis and classification accuracy of brain tumors from MRI scans.

## Methods

This diagnostic study was approved by the Ethics Committee of Beijing Tiantan Hospital in China. Written informed consent was obtained from the patient or the patient’s legally authorized representative.

### Study Participants

This study retrospectively analyzed MRI data of patients with brain tumors collected from 2000 to 2019 at the Beijing Tiantan Hospital in China. The presence of a pathologically confirmed, single brain tumor with the availability of pretreatment MRI scans were the inclusion criteria. Patients with more than 1 type of tumor, poor quality MRI scans, or medically reported conditions that mimic tumors (eg, infarct, infection, and demyelination) or without written informed consent were excluded. For each patient, multiple anisotropic MRI sequences including T1-weighted images (T1WI), T2-weighted images (T2WI), and T1-weighted contrast-enhanced images (T1C) were acquired. More details about scanner types and sample distributions are presented in eTable 1 in the Supplement. This study included 18 types of CNS tumors, which were defined according to World Health Organization (WHO)^19^ standards. Tumors were redefined according to 2016 WHO criteria after screening and rechecking the data. The list of all tumors, along with their imaging definitions and example images, are presented in eFigure 1 and eTable 2 in the Supplement. Selected tumor subtypes were chosen based on the frequency of their occurrence in clinical environments.

The ground truth tumor lesion mask and types were labeled as follows: The lesion masks were labeled by 50 technicians based on MRI scans, pathological reports, and electronic health records of the patient. Two neuroradiologists with more than 18 years of experience evaluated the quality of the labeled mask. Furthermore, for one-quarter of the randomly selected included patients (9468 patients [25.0%]), the exact tumor region was marked on MRI scans for precise localization of the tumor region. Details of this labeling process are presented in eMethods 1 in the Supplement.

To independently test automated tumor classification performance of the developed system, 1 internal and 3 external test data sets were collected with the previously described protocol from Beijing Tiantan Hospital, Jilin University-Affiliated Hospital, Shanxi Hospital, and 301 Hospital. Furthermore, to evaluate whether aid of the proposed DLS was associated with improved diagnostic accuracy of neuroradiologists, a separate internal test data set was collected from Beijing Tiantan Hospital. The ground truth diagnosis for test data sets was performed by 2 neuroradiologists using MRI scans, pathological reports, and electronic health records of patients.

### Development of the DLS for Tumor Segmentation and Classification

For identification and accurate classification of brain tumors, a 2-staged DLS was developed. The complete network architecture is presented in eFigure 2 in the Supplement. Stage 1 of the architecture was designed as a 2-dimensional tumor segmentation network, which segmented regions associated with all 18 types of tumors using 2-dimensional axial slices of MRI sequences. After segmentation, stage 2 of the DLS was designed as a classification network; using the tumor prediction mask from stage 1 and the original MRI scans, this network classified the identified tumor in 1 of the 18 classes. The stage 1 segmentation network was trained using data collected with tumor segmentation masks, whereas the classification network was trained with 75% of the training data. The remaining 25% of the data were reserved as an internal validation set used during the training process for model optimization and hyperparameter selection. The overall distribution of the training data is presented in eFigure 3 in the Supplement, and details of DLS training are presented in eMethods 2 in the Supplement. In this manner, the trained DLS was used to segment and classify tumors in all patients from test data sets.

In the 4 independent test data sets, the tumor classification accuracy of the proposed DLS was compared with the diagnostic accuracy of neuroradiologists with 9 to 30 years of clinical experience (2-3 neuroradiologists from each hospital). For evaluation of improvements in neuroradiologist tumor classification accuracies, 9 neuroradiologists with 5 to 20 years of experience were asked to classify the tumor first without and next with assistance of DLS predictions. Data that support findings of this study are available from the corresponding author upon reasonable request.

### Statistical Analysis

The tumor classification performance of the DLS was evaluated by plotting receiver operating characteristic (ROC) curves and calculating area under the ROC (AUROC) values. The performance was further assessed via classification accuracy, sensitivity, and specificity.

The difference in tumor classification performance between the DLS and neuroradiologists was compared using the mean accuracy, sensitivity, and specificity and was statistically assessed using a binomial test. F1 scores for all tumor types were also calculated. The 95% CIs were computed based on the Wilson score interval formula, with continuity correction applied as required.

The change in neuroradiologist diagnostic accuracy with vs without the assistance of the DLS was the primary outcome, and it was statistically assessed using a 1-sample exact *t* test. A *P* value < .05 was considered statistically significant. All computations and statistical analyses were performed using the scikit-learn module version 0.22 in the Python 3 programming language version 3.22 (Python Software Foundation). Data were analyzed from March 2019 through February 2020.

## Results

### Study Participants

Among 37 871 patients (mean [SD] age, 41.6 [11.4] years; 18 519 women [48.9%]) included in the training data set, data from 11 716 patients were labeled with exact tumor masks and used for training the segmentation model. The distributions of patient age and tumor types in the training data set are presented in eTables 3, 4, and 5 in the Supplement. Four test data sets included a total of 1339 patients (Beijing Tiantan Hospital: 300 patients; Jilin University Affiliated Hospital: 71 patients; Shanxi Hospital: 99 patients; and 301 Hospital: 869 patients). The internal test data set collected for the evaluation of DLS assistance included 1166 patients. Demographic characteristics of these data sets are presented in Table 1, and the Figure is a patient flow diagram. Additionally, the distribution of tumor classes in data sets is presented in eTable 6 in the Supplement. The MRI manufacturer distribution in test data sets is given in eTable 7 in the Supplement.

**Table 1.** Test Set Evaluation of Deep Learning System

	Training set (N = 37 871)	Pathology confirmed data sets, outcome (95% CI)			
		Tiantan Hospital (n = 300)	Jilin University-Affiliated Hospital (n = 71)	Shanxi Hospital (n = 99)	301 Hospital (n = 869)
Tumor classification, No.	18	18	12	12	16
Images, No.	2 693 856	21 288	2832	5062	56 520
Sex, No (%)					
Women	19 186 (50.66)	145 (48.33)	36 (50.70)	66 (66.67)	498 (57.31)
Men	18685 (49.34)	155 (51.67)	35 (49.30)	33 (33.33)	371 (42.69)
Age, mean (SD), y	41.58 (11.4)	35.97 (13.1)	49.38 (14.2)	51.56 (16.8)	38.72 (18.1
Accuracy, %	NA	73.00 (67.7-77.7)	74.20 (62.1-83.4)	81.20 (70.4-88.6)	73.60 (70.5-76.4)
Sensitivity	NA	0.889 (0.853-0.924)	0.727 (0.621-0.834)	0.876 (0.810-0.942)	0.735 (0.619-0.553)
Specificity	NA	0.963 (0.942-0.984)	0.849 (0.763-0.935)	0.968 (0.93-1.00)	0.941 (0.925-0.985)
Precision	NA	0.747 (0.698-0.795)	0.688 (0.577-0.799)	0.912 (0.857-0.969)	0.644 (0.613-0.674)
F1 score	NA	0.796 (0.751-0.841)	0.618 (0.502-0.735)	0.887 (0.824-0.951)	0.545 (0.513-0.577)
Recall	NA	0.889 (0.853-0.924)	0.727 (0.621-0.834)	0.876 (0.810-0.942)	0.735 (0.619-0.553)

Abbreviation: NA, not applicable.

**Figure.**
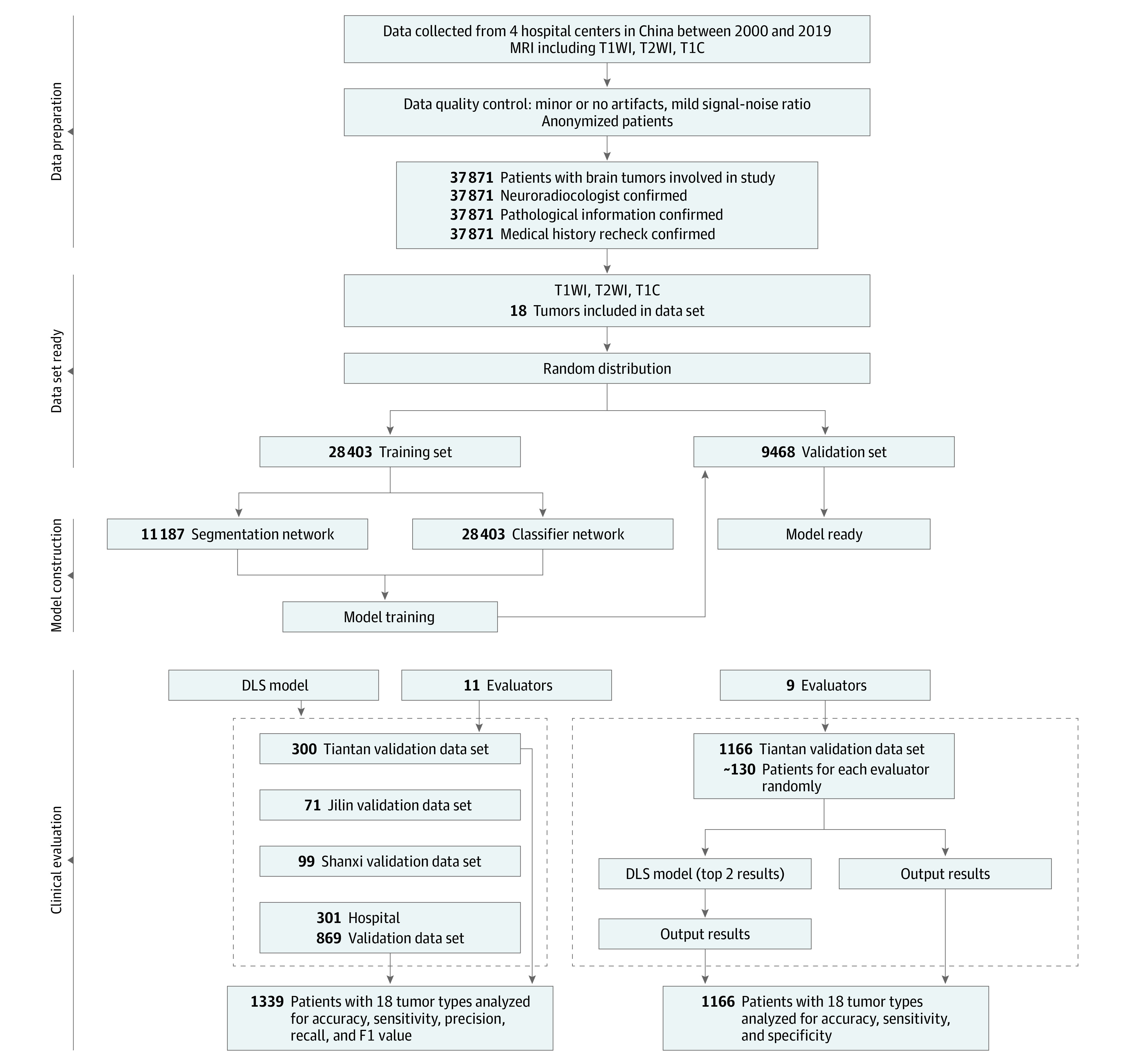
Study Design Overview DLS, deep learning system; MRI, magnetic resonance imaging; T1C, T1-weighted contrast-enhanced images; T1WI, T1-weighted images; T2WI, T2-weighted images.

### DLS for Automated Diagnosis of Brain Tumors and Comparison With Neuroradiologists

On the test data set of 300 patients from Beijing Tiantan Hospital, DLS achieved an 18-class tumor classification accuracy of 73.3% (95% CI, 67.7%-77.7%), which was 19.9% higher than the accuracy of 60.9% (95% CI, 46.8%-75.1%) achieved by neuroradiologists (Table 2). The sensitivity and specificity achieved by the DLS were 88.9% (95% CI, 85.3%-92.4%) and 96.3% (95% CI, 94.2%-98.4%), respectively (Table 1), which were different from the sensitivity and specificity of 53.4% (95% CI, 41.8%-64.9%) and 97.9% (95% CI, 97.3%-98.5), respectively, achieved by neuroradiologists. From these numbers, DLS sensitivity was higher than that of neuroradiologists, but DLS sensitivity was similar. Because sensitivity is the number of true positives divided by the number of positives, a higher sensitivity value may be more important for what we were aiming to achieve than specificity. Baseline information and more details of this analysis are presented in Table 1 and Table 3.

**Table 2.** Accuracy of Professional Evaluators vs DLS^a^

Evaluator index	Years of experience, No.	Accuracy, % (95% CI)
A	15	73.0 (68.0-78.0)
B	11	71.3 (66.2-76.5)
C	13	68.3 (63.1-73.6)
D	19	64.7 (59.3-70.1)
E	15	64.7 (59.3-70.1)
F	30	63.0 (57.5-68.5)
G	10	60.3 (54.8-65.9)
H	9	60.0 (54.5-65.5)
I	10	59.7 (54.1-65.2)
M	9	50.7 (45.0-56.3)
N	24	48.7 (43.0-54.3)
Mean	15	60.9 (46.8-75.1)
DLS	NA	73.0(67.7-77.7)

Abbreviations: DLS, deep learning system; NA, not applicable.

^a^ Among 300 patients from Tiantan Hospital.

**Table 3.** Accuracy in DLS-Assistance Evaluation^a^

Evaluator index	Accuracy, % (95% CI)	
	Without DLS assistance	With DLS assistance
A	67.3 (58.3-76.3)	77.9 (69.9-85.9)
B	51.9 (42.4-61.4)	77.4 (69.4-85.3)
C	61.5 (52.2-70.9)	67.3 (58.3-76.3)
D	42.9 (33.4-52.3)	73.3 (64.9-81.8)
E	59.0 (49.6-68.5)	74.3 (65.9-82.6)
F	81.1 (73.7-88.6)	84.9 (78.1-91.7)
G	66.9 (59.9-73.8)	77.0 (70.8-83.2)
H	68.6 (61.7-75.4)	78.3 (72.2-84.4)
I	65.0 (58.1-71.9)	69.9 (63.3-76.6)
Mean	63.5 (60.7-66.2)	75.5 (73.0-77.9)

Abbreviation: DLS, deep learning system.

^a^ Among 1166 patients in independent internal test data set.

The classification performance achieved by the DLS on 4 test data sets is presented in Table 1. In summary, on 3 external test data sets, the DLS achieved a mean accuracy, sensitivity, and specificity of 73.6% (95% CI, 70.5%-76.4%) to 81.2% (95% CI, 70.4%-88.6%), 72.7% (95% CI, 62.1%-83.4%) to 87.6% (95% CI, 81.0%-94.2%), and 84.9% (95% CI, 76.3%-93.5%) to 96.8% (95% CI, 93.0%-100.0%), respectively, depending on data set. The performance of DLS in patients from the 4 data sets was also visualized by a mean AUORC of 0.92 (95% CI, 0.84-0.99), and each brain tumor’s AUROC is presented in eFigure 3 in the Supplement. For each tumor, the DLS achieved a mean diagnosis accuracy (among the top 2 results output by the DLS) of 85.1% (95% CI, 83.1%-87.0%), sensitivity of 84.2% (95% CI, 72.3%-93.2%), and specificity of 99.1% (95% CI, 90.4%-100.0%). The classification performance of DLS for each tumor type is presented in Table 4.

**Table 4.** Top 2 Validation Tests in Multiple Tumors^a^

Tumor type	Patients, No.	Sensitivity (95% CI)	Specificity (95% CI)	Precision (95% CI)	F1 score (95% CI)	Recall (95% CI)
Acoustic neuroma	205	0.89 (0.84-0.93)	0.99 (0.98-1.0)	0.95 (0.90-0.97)	0.92 (0.88-0.94)	0.89 (0.84-0.93)
Pituitary tumor	234	0.90 (0.86-0.94)	0.96 (0.94-0.97)	0.82 (0.77-0.87)	0.86 (0.83-0.89)	0.90 (0.86-0.94)
Epidermoid cyst	21	0.82 (0.61-0.93	1.00 (1.00-1.00)	0.95 (0.75-0.99)	0.88 (0.75-0.95)	0.82 (0.61-0.93)
Meningioma	280	0.93 (0.9-0.96))	0.94 (0.92-0.95)	0.81 (0.76-0.85)	0.87 (0.84-0.89)	0.93 (0.9-0.96)
Paraganglioma	37	0.37 (0.24-0.52	1.00 (0.99-1.00)	1.00 (0.80-1.00)	0.54 (0.41-0.66)	0.37 (0.24-0.52)
Craniopharyngioma	47	0.93 (0.82-0.98)	0.99 (0.99-1.00)	0.82 (0.70-0.90)	0.88 (0.79-0.93)	0.93 (0.82-0.98)
Glioma	262	0.92 (0.87-0.95)	0.98 (0.97-0.99)	0.91 (0.86-0.94)	0.91 (0.88-0.93)	0.92 (0.87-0.95)
Hemangioblastoma	27	0.88 (0.70-0.96)	1.00 (0.99-1.00)	0.92 (0.74-0.98)	0.90 (0.78-0.96)	0.88 (0.70-0.96)
Metastatic tumor	44	0.74 (0.59-0.85)	0.99 (0.90-0.99)	0.72 (0.57-0.83)	0.73 (0.63-0.81)	0.74 (0.59-0.85)
Germ cell tumor	24	0.67 (0.47-0.82)	1.00 (0.99-1.00)	0.73 (0.52-0.87)	0.70 (0.55-0.81)	0.67 (0.47-0.82)
Medulloblastoma	25	0.86 (0.67-0.95)	1.00 (1.00-1.00)	0.95 (0.76-0.99)	0.90 (0.78-0.96)	0.86 (0.67-0.95)
DNET	14	0.64 (0.38-0.84)	1.00 (0.99-1.00)	1.00 (0.70-1.00)	0.70 (0.46-0.94)	0.64 (0.38-0.84
Chordoma	22	0.86 (0.67-0.95)	1.00 (1.00-1.00)	0.95 (0.76-0.99)	0.90 (0.78-0.96)	0.86 (0.67-0.95)
Lymphomas	43	0.56 (0.41-0.71)	0.99 (0.99-1.00)	0.85 (0.66-0.94)	0.68 (0.56-0.77)	0.56 (0.41-0.71)
Choroid plexus papilloma	18	0.61 (0.39-0.80)	1.00 (0.99-1.00)	0.85 (0.58-0.96)	0.71 (0.53-0.84)	0.61 (0.39-0.80)
Gangliocytoma	13	0.50 (0.25-0.75)	0.99 (0.99-1.00)	0.40 (0.20-0.64)	0.44 (0.28-0.63)	0.50 (0.25-0.75)
Hemangiopericytoma	13	0.44 (0.19-0.73)	1.00 (0.99-1.00)	0.50 (0.22-0.78)	0.47 (0.26-0.69)	0.44 (0.19-0.73)
Other	11	0.25 (0.09-0.53)	1.00 (98-1.00))	0.6 (0.23-0.88)	0.35 (0.17-0.59)	0.25 (0.09-0.53)

Abbreviation: DNET, dysembryoplastic neuroepithelial tumor.

^a^ Top 2 results output by the deep learning system among 1339 patients from 4 centers.

### Association of DLS With Diagnostic Accuracy of Neuroradiologists

To test the association of the DLS with neuroradiologist diagnosis performance, we tested in a routine clinical environment using the internal test data set of 1166 patients. This data set was divided into 9 sets containing approximately 130 patients each; those 9 sets were assessed by 9 neuroradiologists (1 set per neuroradiologist). The neuroradiologists performed diagnosis of the tumors based on MRI characteristics only. Next, the data were again shuffled and the neuroradiologists performed diagnosis of the tumors with the availability of DLS prediction results as a reference. No neuroradiologist viewed the same data twice after randomization.

In this analysis, the performance of the DLS, neuroradiologist without assistance of DLS, and neuroradiologist with assistance of DLS are presented as ROCs in eFigure 3 in the Supplement. The detailed performance of neuroradiologists with and without DLS assistance is presented in Table 3. Neuroradiologists without DLS assistance achieved a mean accuracy of 63.5% (95% CI, 60.7%-66.2%), sensitivity of 63.8% (95% CI, 61.1% 65.8%), and specificity of 95.3% (95% CI, 94.2% 96.1%) in diagnosis of 18 tumor classes. When neuroradiologists had access to DLS results and made necessary modifications and corrections to their diagnoses, the accuracy increased to 75.5% (95% CI, 73.0%-77.9%), sensitivity to 81.4% (95% CI, 78.3% 83.2%), and specificity to 97.1% (95% CI, 96.4%-97.4%). The accuracy increase was 18.9%.

To further analyze this improvement in diagnostic accuracy, based on the results of neuroradiologists without the assistance of DLS analysis, the entire test data set was subdivided into conflict and agreement subsets. The former consisted of diagnoses in which 1 among the DLS or evaluators was wrong but the other was correct. The latter consisted of diagnoses in which the DLS and neuroradiologist were both wrong (209 diagnoses [17.9%], consisting mostly of gangliocytoma, dysembryoplastic neuroepithelial tumor, hemangiopericytoma, lymphoma, medulloblastoma, and glioma and its subtypes) (eFigure 4 in the Supplement) or both correct (631 diagnoses [54.1%]) (eFigure 3 in the Supplement). In the conflict set, when evaluators alone made the wrong diagnosis, they could choose to correct their decisions after referring to DLS results in the analysis of neuroradiologists with the assistance of DLS. In this analysis, 332 diagnoses (28.5% of total diagnoses) were observed to be present in the conflict subset; of these, DLS was correct in 219 diagnoses (66.0% of the conflicting subset) and neuroradiologists were correct in 113 diagnoses (34.0%). In these patients, when neuroradiologists were assisted by the DLS, they corrected their diagnoses in 166 patients (50.0% of the conflicting subset), whereas the DLS suggestion was ignored by neuroradiologists in the remaining 53 patients (16.0% of the conflicting subset). The incorrectly uncorrected diagnoses primarily included tumors of type hemangiopericytoma (24 patients [7.2%]), lymphoma (20 patients [6.0%]), medulloblastoma (4 patients [1.2%]), pituitary adenoma (3 patients [0.9%]), and glioma and its subtypes (2 patients [0.6%]).

## Discussion

In this diagnostic study, a robust DLS for automated segmentation and classification of 18 types of brain tumors from MRI scans was proposed. The system was developed with a large training data set of 37 871 patients, and its generalizability in automated tumor classification was tested with 1 internal and 3 external data sets containing a total of 1339 patients. Results suggested that DLS matched or surpassed experienced neuroradiologists in the identification and classification of intracranial tumors. In the automated classification of tumors, the DLS outperformed experienced neuroradiologists by achieving 19.9% higher classification accuracy (60.9% vs 73.3%). Additionally, the clinical utility of the system in assisting neuroradiologists in tumor diagnosis was evaluated using another independent internal test data set of 1166 patients. With assistance of the proposed DLS, there was a 18.9% increase in neuroradiologist tumor classification accuracy (from 63.5% to 75.5%). To our knowledge, this is 1 of the first studies presenting the development and validation of a clinically viable system for the automated and assisted diagnosis of 18 types of brain tumors.

The task of segmentation of brain tumors from MRI scans has been attempted by several studies in the literature.^20,21,22,23,24,25^ However, our study was larger than those previously carried out, and we validated our algorithm differently. To our knowledge, this is the first large study using deep learning on multiple types of brain tumors (18 types) that contains a large number of training diagnoses. The findings suggest that deep learning algorithms may be able to perform this task with high accuracy. In this study, algorithm validation was unique. We aimed to not only build a reliable algorithm for detecting brain tumors, but also explore the clinical utility of this system in assisting neuroradiologists. We conducted 2 tests to evaluate performance of the DLS under different circumstances. In the first experiment, the DLS was assessed using a data set with approximately equal distributions of tumor types to avoid statistical bias. The purpose of this test was to compare performance of the DLS with that of human evaluators. The second experiment examined the DLS with a data set that followed a natural distribution of brain tumor types; its purpose was to investigate whether human evaluators could improve their diagnostic accuracy with the assistance of the DLS. These experiments complemented each other, and their findings suggest the practicality of applying a DLS in the clinical context.

In the evaluation of the DLS for automated diagnosis of brain tumors, the DLS achieved high accuracy in all test data sets. In particular, the accuracy of DLS was higher than the mean accuracy of all experienced neuroradiologists. These results suggest that the DLS may be able to achieve accuracies comparable with or even higher than those of experienced neuroradiologists in the diagnosis of brain tumors. Furthermore, the DLS may also be able to perform diagnoses more quickly than evaluators.

In the DLS-assistance evaluation, a different experiment set was used to investigate how the DLS may aid neuroradiologists in making diagnostic decisions. In this data set, neuroradiologists and the DLS diagnosed tumors separately, and then neuroradiologists made diagnoses again with DLS assistance. In this way, neuroradiologists could use DLS results as a reference in an attempt to increase overall diagnostic accuracy. Findings suggest that the DLS may be integrated into clinical workflows and aid neuroradiologists in making more accurate diagnoses. The core objective for developing this DLS was to help neuroradiologists make diagnoses more accurately. Of course, sometimes the DLS system may mislead neuroradiologist to make the wrong diagnosis. We investigated these diagnoses with the physicians. The feedback was that they were not confident in their diagnoses because there was not enough information or distinctive characteristics in images. Through big data training, the algorithm used in this study achieved a satisfactory accuracy level, suggesting its potential as a valuable tool in clinical practice.

The traditional paradigm for patients with brain tumors entails an initial radiologic diagnosis of the lesion, which is followed by a therapeutic schedule based on clinical factors and surgeon or patient preferences. For tumors, a definitive histopathologic diagnosis could be obtained only after performing a biopsy and molecular genotyping at centers with such resources.^2^ This process is generally time consuming. With a DLS, clinicians may have the opportunity to determine the tumor type preoperatively and rapidly, shortening the time required for therapy and gaining precious time for treatment, which is associated with improved prognosis. Moreover, in locations around the globe with limited access to expert neuroradiologists, the DLS may offer the advantage of adaptability given that it was developed using extensive CNS tumor data.

### Limitations

This study has several limitations. All training data were obtained from a single center, and training with multicenter, multiethnicity data may be associated with a more generalizable, robust, and reliable system. The number of rare tumors was relatively lower in the training data set, and this class imbalance may impart statistical bias in model performance. Additionally, the proposed system was trained only with axial MRI slices. Therefore, the inclusion of different MRI views and addition of clinical information in predictive modeling may be associated with improved performance of the system. In the future, the proposed system may be extended to aid in treatment planning and for the prognosis of functional outcomes.^7,9^

## Conclusions

This diagnostic study presented an automated, consistent, reliable, and robust system that diagnosed and classified 18 types of brain tumors with high accuracy and aided neuroradiologists in making diagnoses. These outcomes suggest that the DLS may be able provide more accurate information for medical care and eventually constitute a step toward improved tumor diagnoses.

## Notes

Supplement. **eMethods 1.** Annotation of Tumors in Training Data **eMethods 2.** Development of Deep Learning System (DLS) **eFigure 1.** Classification of Brain Tumors Included in Study **eFigure 2.** Structure of Deep Learning System Used for Brain Tumor Detection and Diagnosis **eFigure 3.** Performance of Deep Learning System **eFigure 4.** Neuroradiologist Diagnosis Performance With or Without Deep Learning System Assistance by Tumor Type **eTable 1.** Magnetic Resonance Imaging Sequence Parameters in Training Data **eTable 2.** Definitions of Imaging Characteristics for Multiple Brain Tumors **eTable 3.** Age Distribution of Patients in Training Data Sets **eTable 4.** Age Distribution of Patients in All Test Data Sets **eTable 5.** Tumor Type Statistics in Training Data Sets **eTable 6.** Brain Tumor Distribution in All Data Sets **eTable 7.** Magnetic Resonance Imaging Manufacturer Distribution in All Test Data Sets
