# Supplementary material for: Development and Validation of a Deep Learning Model for Brain Tumor Diagnosis and Classification Using Magnetic Resonance Imaging
Source: JAMA Netw Open. 2022 Aug 8;5(8):e2225608. doi: 10.1001/jamanetworkopen.2022.25608 (PMC9361083; doi:10.1001/jamanetworkopen.2022.25608)
Supplement: Supplement. — eMethods 1. Annotation of Tumors in Training Data eMethods 2. Development of Deep Learning System (DLS) eFigure 1. Classification of Brain Tumors Included in Study eFigure 2. Structure of Deep Learning System Used for Brain Tumor Detection and Diagnosis eFigure 3. Performance of Deep Learning System eFigure 4. Neuroradiologist Diagnosis Performance With or Without Deep Learning System Assistance by Tumor Type eTable 1. Magnetic Resonance Imaging Sequence Parameters in Training Data eTable 2. Definitions of Imaging Characteristics for Multiple Brain Tumors eTable 3. Age Distribution of Patients in Training Data Sets eTable 4. Age Distribution of Patients in All Test Data Sets eTable 5. Tumor Type Statistics in Training Data Sets eTable 6. Brain Tumor Distribution in All Data Sets eTable 7. Magnetic Resonance Imaging Manufacturer Distribution in All Test Data Sets [file jamanetwopen-e2225608-s001.pdf]

Supplementary Online Content

Gao P, Shan W, Guo Y, et al. Development and validation of a deep learning model for brain tumor diagnosis and classification using magnetic resonance imaging. *JAMA Netw Open*. 2022;5(8):e2225608. doi:10.1001/jamanetworkopen.2022.25608

eMethods 1. Annotation of Tumors in Training Data
eMethods 2. Development of Deep Learning System (DLS)
eFigure 1. Classification of Brain Tumors Included in Study
eFigure 2. Structure of Deep Learning System Used for Brain Tumor Detection and Diagnosis
eFigure 3. Performance of Deep Learning System
eFigure 4. Neuroradiologist Diagnosis Performance With or Without Deep Learning System Assistance by Tumor Type
eTable 1. Magnetic Resonance Imaging Sequence Parameters in Training Data
eTable 2. Definitions of Imaging Characteristics for Multiple Brain Tumors
eTable 3. Age Distribution of Patients in Training Data Sets
eTable 4. Age Distribution of Patients in All Test Data Sets
eTable 5. Tumor Type Statistics in Training Data Sets
eTable 6. Brain Tumor Distribution in All Data Sets
eTable 7. Magnetic Resonance Imaging Manufacturer Distribution in All Test Data Sets

This supplemental material has been provided by the authors to give readers additional information about their work.

© 2022 Gao P et al. JAMA Network Open.

eMethods 1. Annotation of Tumors in Training Data

For the precise localization of tumor regions, the tumors were labeled on the MRI scans under the supervision of neuroradiologists. The tumor and cyst areas were annotated using a single binary annotation label. Labeling was done on the T2 axial volume, and T1 contrast- enhanced axial volume.

© 2022 Gao P et al. JAMA Network Open.

eMethods 2. Development of Deep Learning System (DLS)

Design and training of the Deep Learning System

To detect and classify 18 different types of tumors, a two-staged deep learning system (DLS) was designed. Stage one of the DLS consisted of a segmentation network that segmented the tumor regions from the healthy tissue and the second stage classified the identified tumor into one of the 18 classes. This complete architecture of the DLS is presented in supplemental figure S2.

Before the processing with the DLS, all the available 3D MRI scans were preprocessed with intensity normalization to have zero mean and unit standard deviation. Next, the scans were spatially normalized with bilinear interpolation to have uniform axial slice dimensions of 256x256 pixels. Considering the high pixel spacing along the axial direction which would result in poor interpolation performance, the images were not interpolated along the axial direction and an original number of axial slices was retained. Also, when using data from multiple MRI sequences, no head alignment, skull stripping, or registration to a standard brain template was performed to avoid the introduction of any potential registration errors and to reduce the processing time.
The first stage of the DLS was designed as a modified 2D U-Net1 architecture that performed a binary segmentation of tumor regions from the 2D axial slices of the preprocessed MRI sequences. The complete 3D tumor segmentation was obtained by concatenating the 2D predictions from all axial slices. As presented in supplemental figure S2, the modified UNet architecture used in this work consisted of 4 down-sampling and corresponding 4-up-sampling convolution blocks. This stage of the DSL was trained with 30% of the training data (N = 11,716) for which the tumor regions were manually annotated. Of this data, 80% was used for segmentation network training and the remaining 20% was reserved for the internal testing of the segmentation network. With this data, the segmentation network was trained using Adam optimizer for 100 epochs with the dice loss function and an initial learning rate of 10-3. The learning rate was reduced by a factor of two if the dice loss on the internal validation set (20% of the segmentation training set) did not decrease for 10 consecutive epochs. The model with the best loss on the internal segmentation validation set was selected as a final segmentation model.

Stage two of the DLS, the classification network, was designed using 5 modified DenseNet2 blocks and it classified tumors into 18 classes using the 3D MRI scans and the segmentation network's output. The classification network accepted the 3D MRI scans of size 24x256x256 and produced the class probability of each of the tumor classes as an output. In cases where the number of axial slices in the MRI data was less than 24, the input scans were zero-padded to 24 slices. Whereas, for patients with more than 24 slices, data of 24 slices that included the tumor region at the center were analyzed. The classification network was trained using the entire training data with Adam optimizer for 100 epochs the cross-entropy loss function and an initial learning rate of 10-3. Similar to the segmentation network training, the learning rate was reduced by a factor of two if the loss on the internal validation set (20% of the complete training set) did not decrease for 10 consecutive epochs.

© 2022 Gao P et al. JAMA Network Open.

The model with the best loss on the internal validation set was selected as a final classification model.

Moreover, in our dataset, as is the case with the real-world clinical data, all MRI series were not always available for all the patients. Therefore, to accommodate this real-world scenario, using available series from all the patients, we trained a total of 6 architecturally identical models that accepted either a single MRI sequence (T1WI, T2WI, T1C) or a combination of 2 MRI sequences stacked as an input (T1WI & T2WI, T1WI & T1C, T2WI & T2C). At the time of testing, for patients with only one available series, the tumor class predicted by the corresponding series model was considered as a final prediction. For patients with two available series, the majority class predicted by the corresponding 3 models (2 single series models and one combined series model) was considered as a final prediction. For three series scenario, a majority voting was performed on the predictions of all 3 combined series models to determine the final predicted class.

Reference:

1. Ronneberger O, Fischer P, Brox T. U-Net: Convolutional Networks for Biomedical Image Segmentation. Lecture Notes in Computer Science Medical Image Computing and Computer-Assisted Intervention – MICCAI 2015. 2015:234-241. doi:10.1007/978-3- 319-24574-4_28.

2. Huang G, Liu Z, Maaten LVD, Weinberger KQ. Densely Connected Convolutional Networks. 2017 IEEE Conference on Computer Vision and Pattern Recognition (CVPR). 2017. doi:10.1109/cvpr.2017.243.

© 2022 Gao P et al. JAMA Network Open.

eFigure 1. Classification of Brain Tumors Included in Study

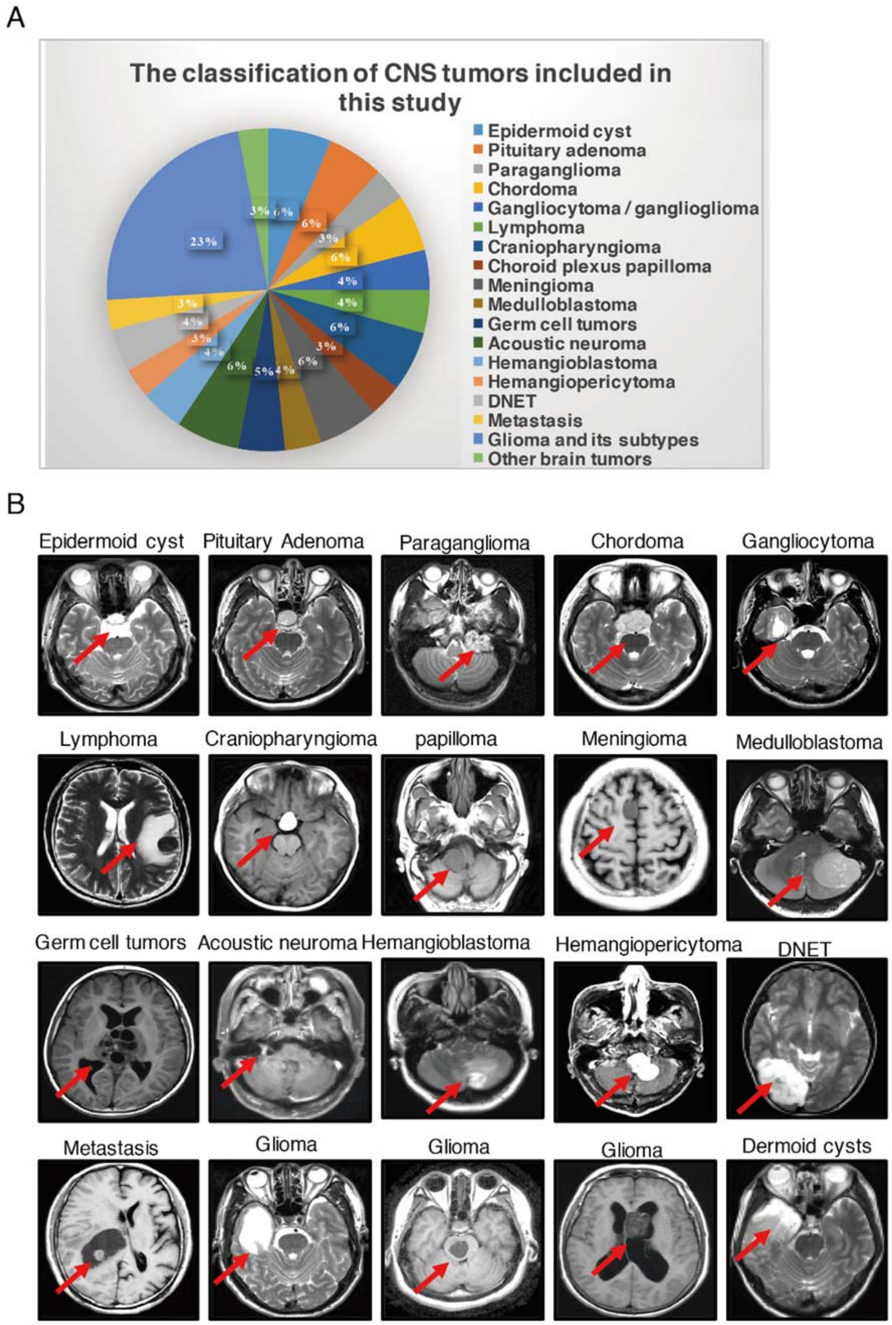

eFigure 1. Classification of brain tumors included in this study.

(A): List of all the 18 tumor types considered in this study and the distribution of these tumor types in the training dataset.

(B): Representative MRI scans of some of the included tumor types and variations between them.

(Abbreviations: DNET: Dysembryoplastic neuroepithelial tumor)

© 2022 Gao P et al. JAMA Network Open.

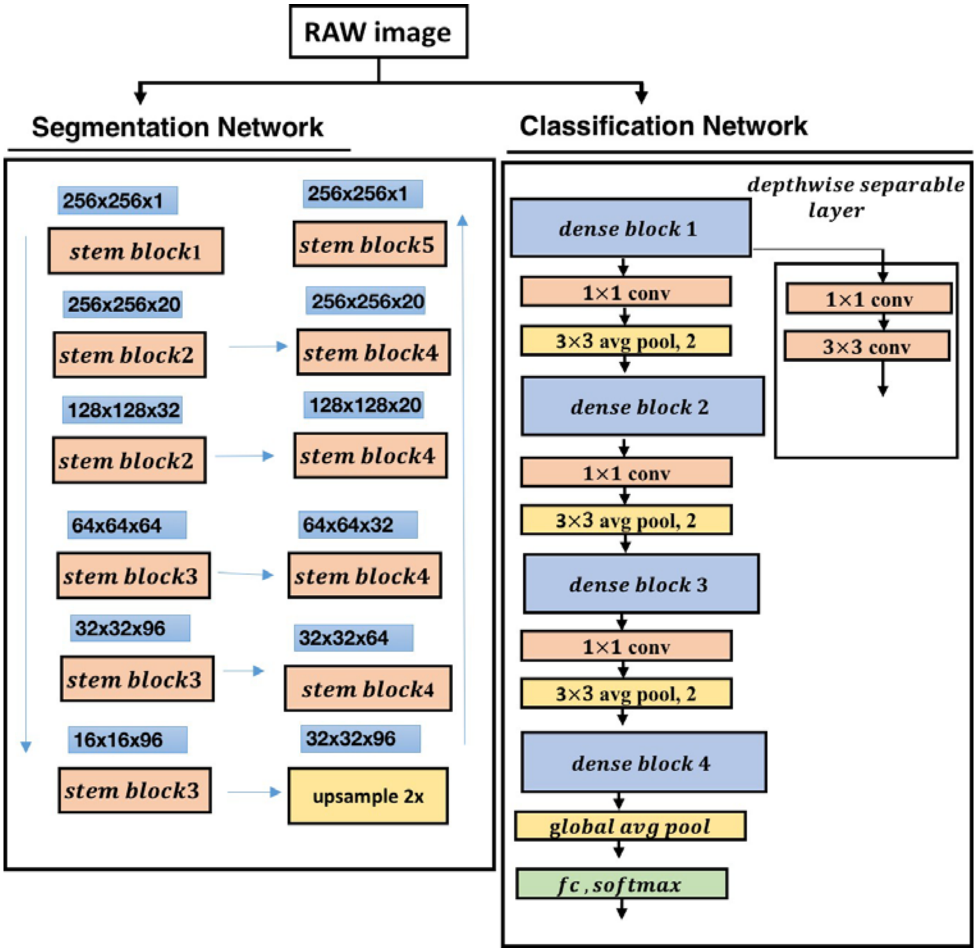

eFigure 2. Structure of Deep Learning System Used for Brain Tumor Detection and Diagnosis

The structure of the DLS system including the segmentation and classification network. Each encoder block contains one or more convolution steps followed by max-pooling and down-sampling processes. Each time the feature maps are down-sampled, the number of output channels are increased. Each decoder block comprises of one deconvolution (transpose convolution) operation which up-samples the size of the feature maps and correspondingly reduces the number of output channels.

© 2022 Gao P et al. JAMA Network Open.

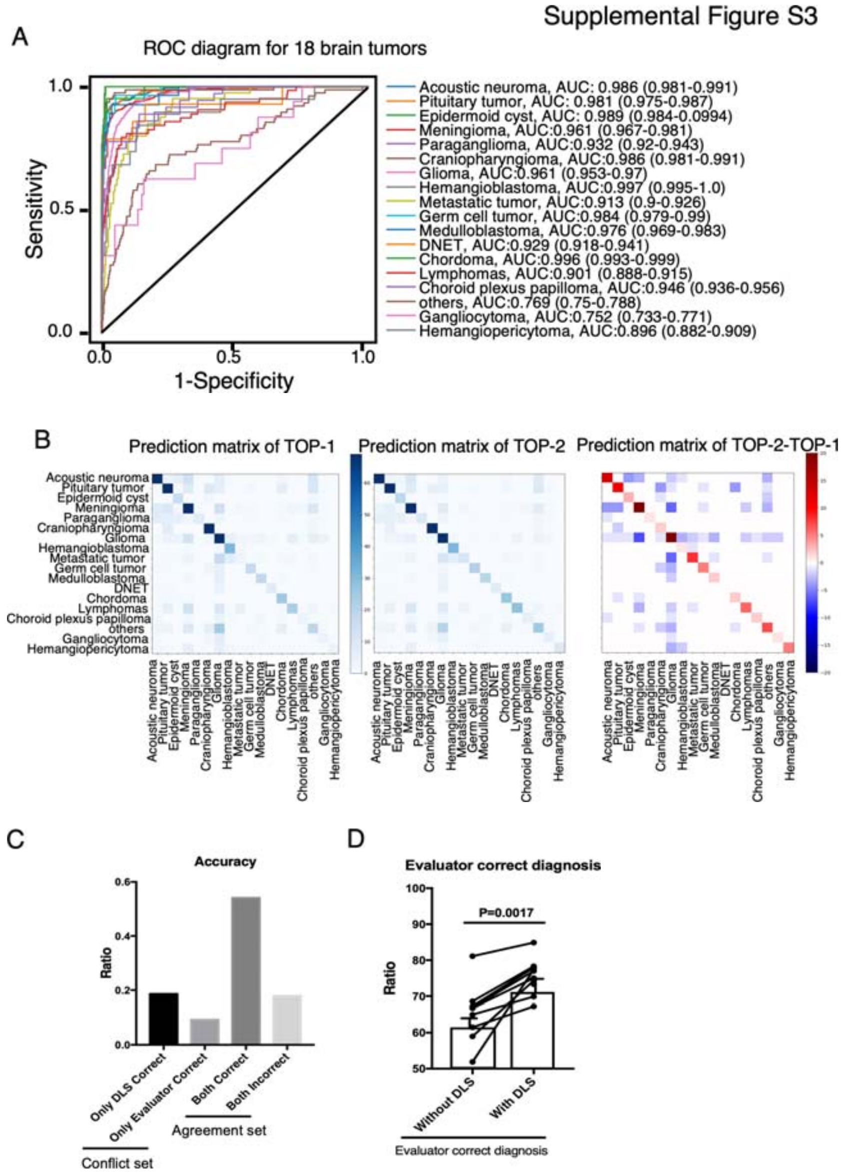

eFigure 3. Performance of Deep Learning System

(A) Receiver operating characteristic (ROC) curves of the deep learning system on the test datasets from Tiantan Hospital with 18 classes (B) Confusion matrix of DLS with Top-1 prediction, Top-2 prediction, and Top-2-Top-1 predictions. (C) The classification accuracy achieved by the neuroradiologists and DLS on the internal test dataset used in the DLS assistance analysis. (D) Example showing when neuroradiologists and DLS disagreed, the DLS helped neuroradiologists correct their prediction in 14% of all the test cases.

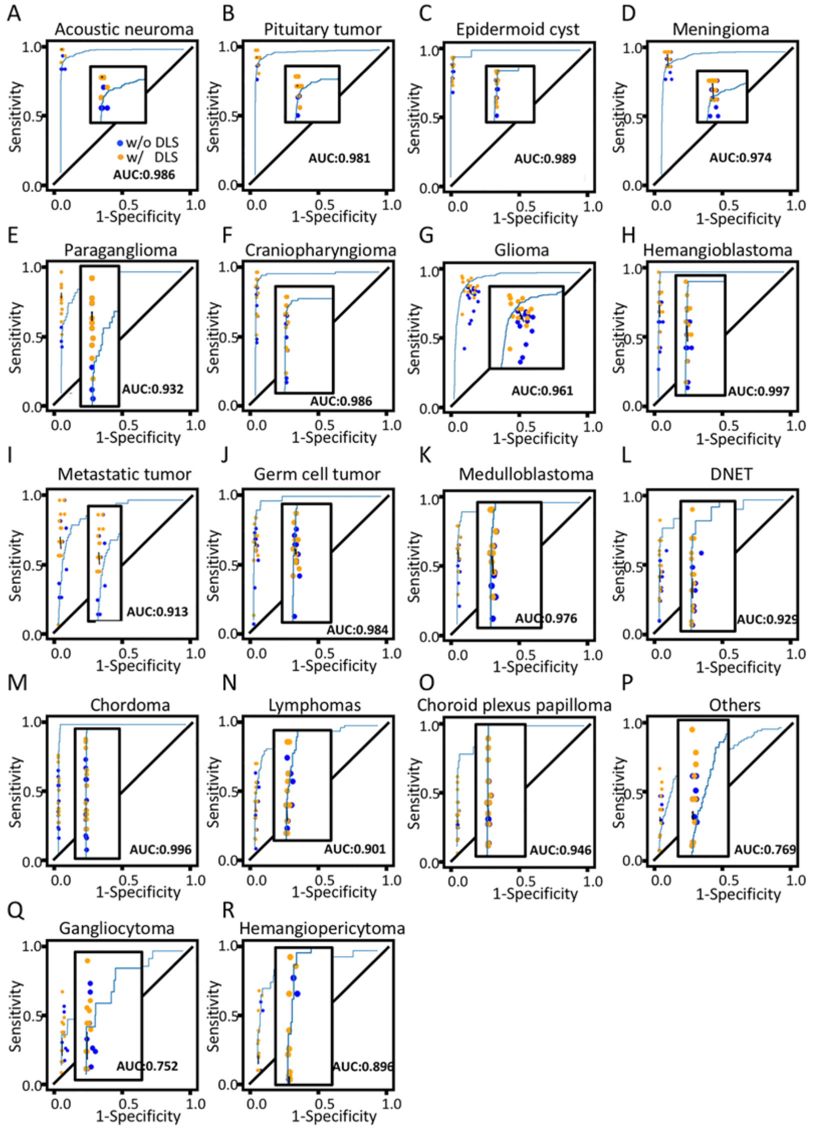

eFigure 4. Neuroradiologist Diagnosis Performance With or Without Deep Learning System Assistance by Tumor Type

(A-R) ROC diagram and enlarged ROC diagram for acoustic neuroma, pituitary tumor, epidermoid cyst, meningioma, paraganglioma, craniopharyngioma, glioma, hemangioblastoma, metastatic tumor, germ cell tumor, medulloblastoma, DNET, chordoma, lymphomas, choroid plexus papilloma, others, gangliocytoma, hemangiopericytoma respectively. The results were calculated from the 1166 patients independent test dataset and confirmed by the patients' pathology results. The blue dots denote the performance of the neuroradiologists without DLS assistance; the yellow dots denote the performance of the neuroradiologists with DLS assistance.

© 2022 Gao P et al. *JAMA Network Open.*

eTable 1. Magnetic Resonance Imaging Sequence Parameters in Training Data

Manufacturer	Model	Thickness (mm)	Resolution (mm*XX/ pixel)
GE Medical Systems	Discovery MR 750	6.0,6.3,6.5	0.5*0.5
GE Medical Systems	Genesis Signa	6.0,6.5,7.0	0.5*0.5
GE Medical Systems	Signa HDe	6.0,6.5,7.0	0.5*0.5
Philips Healthcare	Ingenia	7.0	0.6*0.6/0.4*0.4
Philips Medical systems	Achieva	7.0 or 7.5	0.4*0.4/0.3*0.3
Siemens	Avanto	7.5 -9.8	0.4*0.4/ 0.3*0.3
Siemens	TrioTim	3.3/6.0-9.1/	0.3*0.3/0.5*0.5/0.4*0.4/0.6*0.6/0.7*0.7
Siemens	Verio	6.0-7.2	0.4*0.4/ 0.3*0.3

© 2022 Gao P et al. JAMA Network Open.

eTable 2. Definitions of Imaging Characteristics for Multiple Brain Tumors

Tumors	Classifier			Position
	T1WI	T2WI	T1C	
Acoustic schwannoma	↓	↑ /mix	heterogeneous enhancement	Cerebellopontine Angle
Pituitary tumor	↓	↑	Homogeneous/weak enhancement	hypophyseal fossa
Epidermoid cyst	↓	↑	None	Multiple extracerebral positions
Meningioma	↔	↔	moderate homogeneous enhancement, Dural tail sign	Multiple extracerebral positions
Paraganglioma	↓	↑ Salt and pepper sign	Obvious enhancement	Carotid body/ jugular foramen
Craniopharyngioma	↓ /↔/ ↑	↑ /↔/ ↓	Ring enhancement/calcification	suprasellar
Glioma	↓	↑	None/ weak~obvious enhancement	Braion cortex/subcortical regions
Hemangioblastoma	↓	↑	Mixed cystic and solid lesion/ Obvious nodular enhancement	Infratentorial
Metastatic tumor	↓	↑	Nodular, ring, or punctate enhancement	Braion cortex/subcortical regions
Germ cell tumor	↓	↑	Obvious enhancement	Basal ganglia/sella area/pineal region
Medulloblastoma	↔/ ↓	↔/ ↑	Obvious homogeneous enhancement	Posterior cranial fossa
DNET	↓	↑	None/weak enhancement	Cerebrum cortex/subcortical regions
Chordoma	mixed ↓	mixed ↔/ ↓	Heterogeneous weak enhancement	basilar clivus/midline
Lymphomas	↔/ ↓	↔/ ↓	Homogeneous obvious enhancement	Close to the midline
Choroid plexus papilloma	↔	↔	Homogeneous obvious enhancement/ Mulberry shape	intracerebroventricular
others	N.A	N.A	N.A.	N.A.
Gangliocytoma	↓	↑	None/ weak enhancement	Braion cortex/subcortical regions

© 2022 Gao P et al. *JAMA Network Open.*

Hemangiopericytoma	↓	↑	Obvious enhancement	extracerebral

© 2022 Gao P et al. JAMA Network Open.

eTable 3. Age Distribution of Patients in Training Data Sets

Age	%
0-1	6.7%
1-10	11.7%
10-20	11.8%
20-30	12.3%
30-40	19.0%
40-50	17.6%
50-60	15.4%
60-70	4.4%
70-80	1.0%
80-90	0.1%

eTable 4. Age Distribution of Patients in All Test Data Sets

Age	Tiantan Hospital	Shanxi Hospital	Jilin Hospital	301 Hospital
	(%)	(%)	(%)	(%)
1-10	10.9	-	1.4	1.4
10-20	14.6	-	8.6	5.1
20-30	10.5	2.0	2.9	9.3
30-40	19.9	10.9	10.0	15.8
40-50	21.7	25.7	14.3	26.5
50-60	15.0	24.8	31.4	24.3
60-70	6.7	31.7	27.1	15.0
70-80	0.7	5.0	1.4	2.5
80-90	-	-	2.9	-

© 2022 Gao P et al. JAMA Network Open.

eTable 5. Tumor Type Statistics in Training Data Sets

Index	Tumor type	Number of cases for training	Number of cases for segmentation training	Number of cases for classifier training	Number of cases in validation set	Number of pathologies
1	Epidermoid cyst	1157	885	930	227	525
2	Pituitary adenoma	4433	1163	3329	1104	525
3	Paraganglioma (Glomus jugulare tumor)	51	48	38	13	11
4	Chordoma	792	586	649	143	335
5	Gangliocytoma / ganglioglioma	509	304	423	86	291
6	Lymphoma	693	377	530	163	94
7	Craniopharyngioma	2628	675	1910	718	735
8	Choroid plexus papilloma	317	206	207	110	115
9	Meningioma	5840	514	4168	1672	966
10	Medulloblastoma	716	468	500	216	317
11	Germ cell tumors (including Germinoma and Teratoma)	910	489	583	327	273
12	Acoustic neuroma	3366	891	2404	962	1136
13	Hemangioblastoma	730	433	617	113	305
14	Hemangiopericytoma	293	194	225	68	176
15	Dysembroyoplastic neuroepithelial (DNET)	257	110	183	74	153
16	Metastasis	727	68	609	118	122
17	Glioma	10659	3927	8631	2028	3758
18	Other brain tumors (Including Dermoid cysts, Granular cell tumors, Melanocytic tumors	3793	378	2467	1326	146
	Total	37871	11716	28403	9468	9983

© 2022 Gao P et al. JAMA Network Open.

eTable 6. Brain Tumor Distribution in All Data Sets

		Test Set 1				Test set 2
	Training & validation Set	Tiantan Hospital	Shanxi Hospital	Jilin Hospital	301 Hospital	Tiantan Hospital
1 Epidermoid cyst	1157	19	2	2	0	74
2 Pituitary adenoma	4433	17	27	5	185	64
3 Paraganglioma (Glomus jugulare tumor)	51	10	0	1	27	22
4 Chordoma	792	17	2	1	2	68
5 Gangliocytoma / ganglioglioma	509	12	1	1	0	46
6 Lymphoma	693	13	4	3	23	42
7 Craniopharyngioma	2628	18	2	5	22	74
8 Choroid plexus papilloma	317	10	0	1	8	42
9 Meningioma	5840	18	32	25	205	76
10 Medulloblastoma	716	11	3	1	11	33
11 Germ cell tumors	910	14	1	1	8	56
12 Acoustic neuroma	3366	19	25	2	160	61
13 Hemangioblastoma	730	13	0	1	11	44
14 Hemangiopericytoma	293	9	0	0	2	31
15 Dysembroyoplastic neuroepithelial	257	13	0	0	1	49
16 Metastasis	727	9	0	4	31	42
17 Glioma and its subtypes	10659	69	0	18	169	294
18 Other brain tumors	3793	9	0	0	4	48
Total	37871	300	99	71	869	1166

© 2022 Gao P et al. JAMA Network Open.

eTable 7. Magnetic Resonance Imaging Manufacturer Distribution in All Test Data Sets

Test center	Manufacturer	Model	%
Tiantan Hospital	GE Medical Systems	Signa HDe	21.1
	GE Medical Systems	Genesis Signa	18.8
	GE Medical Systems	Discovery MR 750	8.7
	Siemens	TrioTim	33.9
	Siemens	Verio	15.8
	Toshiba	MRT200F3	1.3
	Unknown	Unknown	0.3
Shanxi Hospital	Siemens	TrioTim	75.7
	Siemens	Avanto	17.4
	GE Medical Systems	Discovery MR 750	7.0
Jilin Hospital	GE Medical Systems	Ingenia	6.3
	Siemens	Avanto	39.2
	Siemens	TrioTim	7.6
	Philips Medical systems	Achieva	27.8
	Philips Medical systems	Ingenia	15.2
	Neusoft	NSM-S15P	2.5
	UIH	uMR 780	1.3
301 Hospital	GE Medical Systems	Signa HDxt	56.5
	GE Medical Systems	DISCOVERY MR750	22.6
	GE Medical Systems	SIGNA EXCITE	4.8
	GE Medical Systems	SIGNA HDxt	4.8
	GE Medical Systems	Optima MR360	1.3
	Siemens	Skyra	0.7
	Siemens	Espree	0.2
	UIH	uMR 770	8.4
	alltech	EchoStar	0.1
	Centauri	Centauri	0.3

© 2022 Gao P et al. JAMA Network Open.
